# Interaction of transactive response DNA binding protein 43 with nuclear factor κB in mild cognitive impairment with episodic memory deficits

**DOI:** 10.1186/2051-5960-2-37

**Published:** 2014-04-01

**Authors:** Yasuyuki Ohta, Cyntia Tremblay, Julie A Schneider, David A Bennett, Frederic Calon, Jean-Pierre Julien

**Affiliations:** 1 Research Centre of Institut universitaire en santé mentale de Québec, Québec, QC, Canada; 2 Department of Psychiatry and Neuroscience, Laval University, Québec, QC, Canada; 3 Research Center of Centre Hospitalier Universitaire de Québec, and Faculty of Pharmacy, Laval University, Québec, QC, Canada; 4 Rush Alzheimer’s Disease Center, Rush University Medical Center, Chicago, IL, USA

**Keywords:** TDP-43, NF-κB, Episodic memory, Mild cognitive impairment, Alzheimer’s disease

## Abstract

**Introduction:**

Transactive response DNA binding protein 43 (TDP-43) is detected in pathological inclusions in many cases of Alzheimer’s disease (AD) and mild cognitive impairment (MCI), but its pathological role in AD and MCI remains unknown. Recently, TDP-43 was reported to contribute to pathogenesis in amyotrophic lateral sclerosis through its interaction with p65 nuclear factor κB (NF-κB) resulting in abnormal hyperactivation of this signaling pathway in motor neurons. Hence, we investigated the interaction of TDP-43 with p65 in the temporal cortex of subjects with a clinical diagnosis of MCI (n = 12) or AD (n = 12) as well as of age-matched controls with no cognitive impairment (NCI, n = 12).

**Results:**

Immunoprecipitation and immunofluorescence approaches revealed a robust interaction of TDP-43 with p65 in the nucleus of temporal lobe neurons in four individuals with MCI (named MCI-p). These MCI-p cases exhibited high expression levels of soluble TDP-43, p65, phosphorylated p65 and low expression levels of β-amyloid 40 when compared to AD or NCI cases. The analysis of cognitive performance tests showed that MCI-p individuals presented intermediate deficits of global cognition and episodic memory between those of AD cases and of NCI cases and MCI cases with no interaction of TDP-43 with p65.

**Conclusions:**

From these results, we propose that enhanced NF-κB activation due to TDP-43 and p65 interaction may contribute to neuronal dysfunction in MCI individuals with episodic memory deficits. Accordingly, treatment with inhibitors of NF-κB activation may be considered for MCI individuals with episodic memory deficits.

## Introduction

Transactive response DNA binding protein 43 (TDP-43) has been implicated in amyotrophic lateral sclerosis (ALS) and frontotemporal lobar degeneration with ubiquitin inclusion (FTLD-U) [1-4]. TDP-43 is a DNA/RNA binding protein regulating gene expression by several processes including gene transcription, RNA splicing, messenger RNA stabilization and transport [5-7]. TDP-43, which is normally found in the cell nucleus, is detected in pathological cytoplasmic inclusions in ALS and FTLD-U [1,3]. Cytoplasmic TDP-43 inclusions have also been reported in combination with classic Alzheimer’s disease (AD) pathology and they are estimated to be present in up to 75% of patients with a pathologic diagnosis of AD [8-12]. The distribution of TDP-43 pathology overlaps with tau pathology in the form of neuropil threads and neurofibrillary tangles (NFT) in AD [13]. Some TDP-43 inclusions in AD were shown to be within neurons with NFT by double immunofluorescence experiments [8]. Interestingly, overexpression of TDP-43 was found to increase activity of β-site amyloid precursor protein (APP) cleaving enzyme 1 (BACE1) enzyme, thereby accelerating APP amyloidogenic metabolism [14]. However, the pathological role of TDP-43 in AD remains unknown.

Mild cognitive impairment (MCI) refers to a transitional state between normal cognition and early dementia, especially AD [15]. MCI is a syndrome defined by clinical, cognitive and functional criteria. Not all MCI cases progress to AD [16]. Biomarkers as diagnostic criteria for MCI due to AD have not yet been established [17]. Although most studies report that levels of β-amyloid (Aβ) and tau pathologies in MCI are intermediate levels between AD and controls [18-20], the neuropathological features of MCI are heterogeneous. A previous study revealed that frequencies of individuals with TDP-43 or phosphorylated TDP-43 cytoplasmic inclusions in the brain were higher in AD cases than age-matched controls with no cognitive impairment (NCI) or than MCI cases at intermediate deficit level [21,22]. However, the pathological involvement of TDP-43 in MCI remains poorly understood.

Recent lines of evidence suggest that in ALS, soluble fractions of TDP-43 interacts with p65 subunit of nuclear factor κB (NF-κB) in the nucleus of neurons and glial cells, and that an upregulation of TDP-43 may contribute to pathogenesis by causing abnormal hyperactivation of p65 NF-κB [23]. These findings led us to examine whether similar phenomena may occur in AD and MCI. It is already known that in AD, the immunoreactivity of p65 NF-κB can be detected in the neuropil of diffuse Aβ deposit and sometimes in the nucleus of subsets of neurons [24]. Moreover, activation of NF-κB may be triggered by β-Amyloid 40 (Aβ40) peptide [25-27], whereas p65 NF-κB expression increases BACE1 activity and APP processing [28,29]. Here, we investigated the interaction of TDP-43 with p65 in the temporal cortex of subjects with MCI and AD as well as in age-matched controls with no cognitive impairment (NCI) using immunoprecipitation and immunofluorescence approaches. Our results revealed an enhanced TDP-43 interaction with p65 in MCI cases exhibiting deficits of global cognition and episodic memory. Accordingly, we propose that hyperactivation of NF-κB may contribute to neuronal dysfunction in the temporal lobe of MCI individuals with episodic memory deficits.

## Materials and methods

### Antibodies

The following antibodies were used in this study: anti-actin (Millipore, Billerica, MA), anti-neuronal nuclear antigen (NeuN) (Chemicon International, Temecula, CA), anti-p65 (Santa Cruz Biotechnology, Santa Cruz, CA), anti-p65 (Invitrogen, Camarillo, CA), anti-phospho-p65 (phosphorylated at serine 536, Cell signaling technology, Danvers, MA), anti-tau 13 (Covance, Princeton, NJ), anti-tau CP-13 (phosphorylated at serine 202/threonine 205, gift from Dr. Peter Davies; Albert Einstein College of Medicine, Bronx, NY), anti-TDP-43 2E2-D3 (human-specific monoclonal antibody to total TDP-43; Abnova, Walnut, CA), and anti-C-TDP-43 (12892 polyclonal antibody to C-terminal TDP-43; ProteinTech Group, Chicago, IL).

### Study participants

Samples from the brain cortex were obtained from participants in the Religious Order Study, a longitudinal clinicopathology study of aging and dementia from which an extensive amount of clinical and neuropathology data were available [30]. The study included participants with the clinical diagnosis of MCI (n = 12), probable AD (n = 12), and persons with no obvious cognitive impairment (NCI, n = 12), as previously described [20]. Dementia and AD diagnosis required evidence of meaningful decline in cognitive function based on the results of 21 cognitive performance tests, which were reviewed by a clinical neuropsychologist and expert clinician. MCI refers to participants with cognitive impairment as assessed by the review of the cognitive performance tests by the neuropsychologist, but without a diagnosis of dementia as assessed by expert clinicians. At death, the clinical diagnosis was reviewed based on all available clinical data by a neurologist blinded to all postmortem data [31-33]. A global measure of cognition was based on 19 cognitive performance tests, which were also used to summarize cognitive abilities in 5 domains: episodic memory, semantic memory, working memory, perceptual speed and visuospatial ability [34].

At death, each case was assigned a Braak score [35] based on neuronal neurofibrillary tangle pathology, a neuritic plaque score based on the modified Consortium to Establish a Registry for Alzheimer Disease (CERAD) criteria [36] and an AD pathologic diagnosis based on the National Institute on Aging – Reagan criteria [37] by examiners blinded to all clinical data, as previously described [18]. After the assessment in Braak, CERAD and Reagan score, 12 subjects with AD, MCI and NCI were selected randomly from participants in the Religious Order Study. Scores of hippocampal atrophy between 0 and 6 (0 = none, 1 = possible, 2 = mild, 3 = mild to moderate, 4 = moderate, 5 = moderate to severe, 6 = severe) were assessed by neuropathologists blinded to all clinical data. Neuritic plaques, diffuse plaques, and neurofibrillary tangles in the inferior parietal cortex were counted after Bielschowsky silver staining, as previously described [38]. Concentrations of Aβ and tau in the temporal and parietal cortex were assessed using enzyme linked immunosorbent assay (ELISA) and Western immunoblotting as described [20].

### Protein lysates preparation and ELISA

For quantification of TDP-43 and p65, postmortem frozen samples (~ 100 mg) from the temporal cortex from 36 study participants were homogenized in a Tris-buffered saline (TBS) consisting of 50 mM Tris-base, 138 mM NaCl, 2.7 mM KCl, protease inhibitor cocktail (Roche, Indianapolis, IN) and phosphatase inhibitor cocktail (Pierce Biotechnology, Rockford, IL), and centrifuged at 20,800 g for 20 min at 4°C. After protein determination was performed by Bradford method (Bio-Rad Laboratories, Hercules, CA), the supernatant was used as a sample (TBS-soluble fraction).

For quantification of total tau and Aβ, postmortem samples from the temporal and inferior parietal cortex from the same 36 study volunteers were homogenized and centrifuged sequentially to generate a TBS-soluble protein fraction and a detergent-insoluble protein fraction (formic acid extract) as described [20]. Tissue samples were homogenized and sonicated 3× for 5 × 1-second pulse in TBS buffer, and centrifuged at 100,000 g for 20 min at 4°C. After removing the supernatant (soluble fraction), the pellet was homogenized and sonicated in lysis buffer (150 mM NaCl, 10 mM NaH2PO4, 0.5% sodium deoxycholate, 0.5% sodium dodecyl sulfate (SDS), 1% Triton X-100, protease inhibitor cocktail and phosphatase inhibitor cocktail, and spin as previously described. After removing the supernatant, the pellet was homogenized in formic acid, sonicated for 10 × 1-second pulse and spin, and the supernatant (insoluble fraction) was removed as previously described. After protein determination was performed, the proteins were dried using a SpeedVac (Thermo Savant, Waltham, MA). The proteins were solublilized in 5 M guanidine for ELISA and in SDS sample buffer (60 mM Tris, 10% glycerol, 2% SDS, 0.0025% bromophenol blue, 2.5% β-mercaptoethanol, pH 8.5) for Western immunoblotting.

Aβ40 and β-Amyloid 42 (Aβ42) concentrations were measured using specific human Aβ ELISA kits (Wako, Osaka, Japan) according to the manufacturer’s recommendations.

### Coimmunoprecipitation and Western blot assays

TDP-43, p65 and tau were quantified using Western immunoblot. Proteins (15 μg/sample) were heated at 95°C for 5 minutes in SDS sample buffer. For immunoprecipitation study, anti-TDP-43 polyclonal (ProteinTech, Chicago) or anti-p65 polyclonal antibody (Santa Cruz Biotechnology, Santa Cruz) was bound to protein G-coated magnetic beads (Dyanl, Invitrogen, Camarillo) and was incubated with 50 μg of lysate overnight at 4°C. After washing, immunoprecipitates were eluted with SDS sample buffer. Samples were resolved by 10% SDS-PAGE and transferred to a PVDF membrane (Polyscreen, PerkinElmer, Boston, MA). The membrane was incubated with anti-p65 (Invitrogen, Camarillo) or anti-TDP-43 2E2-D3 antibody (Abnova, Walnut), and western blot image was obtained using a chemiluminescence detection kit (Pierce Biotechnology, Rockford, IL). Each protein was estimated by standardization with actin.

### Immunofluorescence

Immunofluorescence labeling was performed on 6-μm-thick sections of paraffin-embedded temporal cortex samples from the brains. Before immunostaining, the sections were microwaved 2 × for 2 minutes each in 0.01 mol/L citrate buffer, pH 6.0, for antigen retrieval. Sections were incubated with anti-TDP-43 2E2-D3 (Abnova, Walnut) and anti-p65 antibodies (Santa Cruz Biotechnology, Santa Cruz) and subsequently with corresponding Alexa 488 and 633 antibodies (Molecular Probes, Eugene, OR). The nuclei were counterstained with Dapi (Invitrogen, Camarillo). Sections were observed by confocal laser microscopy (FV300 and FV1000, Olympus, Tokyo, Japan). Autofluorescence, such as lipofuscin pigments, were detected using 575–630 nm bandpass emission filter.

### Data analysis

Data are expressed as means ± SD. Statistical comparisons of data between groups were performed using the *χ*^2^ test or Kruskal-Wallis test, followed by Dunn multiple comparisons test. For comparisons between 2 groups, Mann–Whitney test was performed. Statistical analyses were done using GraphPad Prism 5 (version 5.00; GraphPad Software, Inc., San Diego, CA). Statistical significance was set at *p* < 0.05.

All subjects signed an informed consent and Anatomical Gift Act donating their brain for studies of aging and dementia. The study was approved by the Institutional Review Board of Rush University Medical Center.

## Results

**Figure 1:**
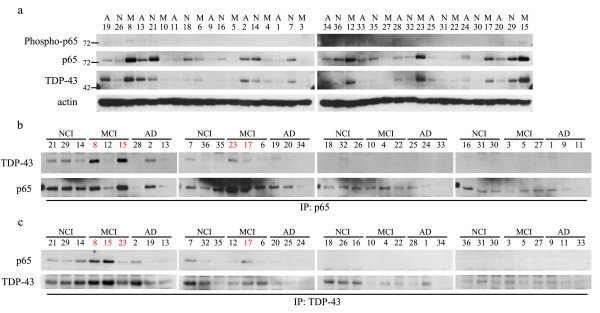
**Full-length TDP-43 interacts with p65 NF-κB in the temporal cortex of four individuals with mild cognitive impairment (MCI). a** The presence of phosphorylated p65 NF-κB at ser536 and accumulation of p65 and full-length of TDP-43 in TBS-soluble fraction from the temporal cortex of individuals with MCI. Protein extracts from the temporal cortex of individuals with no obvious cognitive impairment (NCI; N, n = 12), MCI (M, n = 12) or Alzheimer’s disease (AD; A, n = 12) were subjected to SDS-PAGE and immunoblotting with the indicated antibodies. Actin was used as a loading control. **b**, **c** Interaction of TDP-43 with p65 in TBS-soluble fraction from the temporal cortex of four individuals with MCI (Subjects 8, 15, 17 and 23), one individual with AD (Subject 2) and four individuals with NCI (Subjects 7, 14, 21 and 29). Protein extracts from the temporal cortex of individuals with NCI, MCI or AD were used for immunoprecipitation (IP) with anti-p65 polyclonal antibody **(b)** or anti-TDP-43 polyclonal antibody **(c)**. Immunoprecipitates were subjected to SDS-PAGE and immunoblotting with the indicated antibodies. Number of Subjects 8, 15, 17 and 23 is written in red color.

TBS-soluble fraction of temporal cortex of subjects with NCI, MCI and AD was used for immunoblotting and coimmunoprecipitation experiments. Immunoblotting experiments revealed that the levels of both TDP-43 and p65 were elevated in 5 individuals with MCI (Subjects 8, 12, 15, 17 and 23), 2 individuals with AD (Subjects 2 and 13) and 4 individuals with NCI (Subjects 7, 14, 21 and 29) (Figure 1a). It is established that activation of p65 is associated with its phosphorylation [39]. Moreover, an elevation of phosphorylated p65 at ser536 can be achieved by overexpression of TDP-43 in microglial cells after stimulation with LPS or H_2_O_2_[23]. As shown in Figure 1a, the signal of phosphorylated p65 at ser536 was quite weak in post-mortem tissue of temporal cortex from most subjects. Nonetheless, phosphorylated p65 was detectable by immunoblotting in 4 individuals with MCI (Subjects 8, 12, 15 and 23) and 1 individual with AD (Subject 28). Thus, using χ^2^test, the frequency of positive phospho-p65 were increased in MCI (*χ*^2^ = 6.039, p = 0.049).

To investigate the possible interaction of TDP-43 and p65 in the temporal cortex of subjects with NCI, MCI and AD, we carried out immunoprecipitation assays using anti-TDP-43 polyclonal antibody followed by immunoblotting with anti-p65 monoclonal antibody (Figure 1b). We also carried out the reverse immunoprecipitation using anti-p65 polyclonal antibody followed by immunoblotting with anti-TDP-43 monoclonal antibody (Figure 1c). In both experiments, coimmunoprecipitation of TDP-43 with p65 was detected in 4 individuals with MCI (Subjects 8, 15, 17 and 23), 1 individual with AD (Subject 2) and 4 NCI individuals (Subjects 7, 14, 21 and 29). The most robust interaction of TDP-43 with p65 in both coimmunoprecipitation experiments was detected in brain extracts from individuals with MCI (Subjects 8 and 15) (Figure 1b,c). Both TDP-43 and p65 were highly elevated in 3 individuals with MCI (Subject 8, 15 and 23, TDP-43 and p65 content were 1.2 and 1.3, 1.5 and 1.1, 1.7 and 0.9, respectively) showing the interaction of TDP-43 with p65, but those were mildly elevated in other individuals with MCI, AD and NCI showing this interaction (Figure 1a).

**Figure 2:**
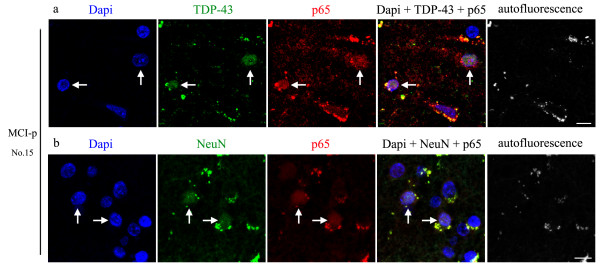
**TDP-43 colocalizes with p65 in the nucleus of neurons of the temporal cortex of individuals with MCI showing the interaction of TDP-43 with p65 in coimmunoprecipitation experiments (Figure**1**B, C; MCI-p). a**, **b** A section from the temporal cortex of MCI-p (Subject 15) were incubated with anti-p65 and anti-TDP-43 **(a)** or anti-NeuN **(b)** antibodies and subsequently with corresponding Alexa 488 and 633 antibodies (Molecular Probes), and imaged by confocal laser microscopy. The nuclei were counterstained with Dapi. Autofluorescence was detected using 575–630 nm bandpass emission filter. Arrows indicate colocalization of Dapi, p65 and TDP-43 **(a)** or NeuN **(b)**. Scale bars, 50 μm.

**Figure 3:**
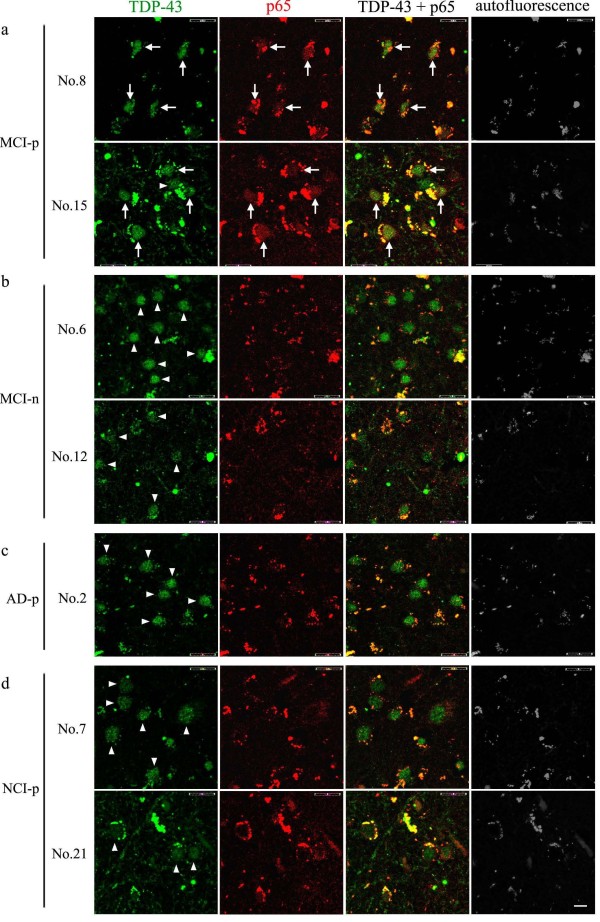
**TDP-43 colocalizes with p65 in the neuronal cells from the temporal cortex of individuals with MCI-p. a**-**d** Sections from the temporal cortex of MCI-p (**a**, Subjects 8 and 15), MCI without the interaction of TDP-43 with p65 in coimmunoprecipitation experiments (Figure 1B, C; MCI-n, **b**, Subjects 6 and 12), AD (**c**, Subject 2) or NCI (**d**, Subjects 7 and 21) showing the interaction of TDP-43 with p65 (Figure 1B,C; AD-p or NCI-p) were incubated with anti-TDP-43 and anti-p65 antibodies and subsequently with corresponding Alexa 488 and 633 antibodies, and imaged by confocal laser microscopy. Autofluorescence was detected using 575–630 nm bandpass emission filter. Arrows indicate the nuclear TDP-43 positive cells colocalized with p65 **(a)**. Arrowheads indicate the nuclear TDP-43 positive cells without colocalization with p65 **(a-d)**. Scale bars, 50 μm.

To examine the subcellular distribution of interaction of TDP-43 with p65, double immunofluorescence staining using anti-TDP-43 and anti-p65 antibodies was performed on paraffin-embedded sections of the temporal cortex from the same series of samples (Figures 2a, 3 and Additional file 1: Figure S1). As expected, TDP-43 was normally found in the nucleus of neuronal cells in the temporal cortex of subjects with NCI, MCI and AD. The nuclear colocalization of p65 with TDP-43 in neurons was observed predominantly in MCI individuals (Figures 2a, 3a and Additional file 1: Figure S1a, arrows) who showed interaction of TDP-43 with p65 as determined by coimmunoprecipitation assays, especially Subjects 8, 15, 17 and 23 that we defined as MCI-p. To identify neuronal cells expressing p65, immunofluorescence staining using anti-p65 plus anti-NeuN antibodies as a neuronal marker was performed for subjects with MCI-p (Figure 2b). Strong signals of p65 were found in the nucleus of many neurons in the subjects with MCI-p (Figure 2b, arrows). On the contrary, subjects with NCI and AD showing the interaction of TDP-43 with p65 in coimmunoprecipitation experiments (Figure 1b,c; Subjects 7, 14, 21 and 29; NCI-p and Subject 2; AD-p) and MCI without the interaction of TDP-43 with p65 (Figure 1b,c; MCI-n) presented only few cells expressing p65 in the nucleus (Figures 3b-d, Additional file 1: Figure S1c). Among the subjects with MCI-p, the frequencies of colocalization of TDP-43 with p65 in the nuclear TDP-43 positive cells were higher in Subjects 8 and 15 (Figure 3a; 45.6%) compared to Subjects 17 and 23 (Additional file 1: Figure S1a; 12.5%). On the contrary, only few TDP-43 positive cells showed p65 signals in the subjects identified as MCI-n (Figure 3b). Note the absence of TDP-43 and/or p65 aggregates in Figures 2 and 3. The cytosolic signals detected are due to autofluorescence. These results corroborated band intensities detected after coimmunoprecipitation of TDP-43 with p65 (Figure 1b,c). Although 1 individual with AD-p (Subject 2) and 4 individuals with NCI-p (Subjects 7, 14, 21 and 29) showed weak interaction of TDP-43 with p65 in coimmunoprecipitation experiments (Figure 1b,c), the frequencies of colocalization of TDP-43 with p65 in TDP-43 cells of these subjects was lower (5.6% and 8.1%, respectively) than MCI-p (mean of Subjects 8, 15, 17 and 23 is 29.0%). In AD without the interaction of TDP-43 with p65 in coimmunoprecipitation experiments (Figure 1b,c; AD-n), only few TDP-43 positive cells showed p65 signals (Additional file 1: Figure S1b). This result suggests that the interaction of TDP-43 with p65 in the neurons of temporal lobe was stronger in some subjects with MCI (Subjects 8, 15, 17 and 23) compared to AD and NCI. In previous work using same series of samples [21], TDP-43 immunofluorescence in the cytoplasm of neuron-like cells was detected in 6 individuals with AD, 4 individuals with MCI and one individual with NCI, which is not a typical pathology as FTLD-TDP [2,40,41].

**Figure 4:**
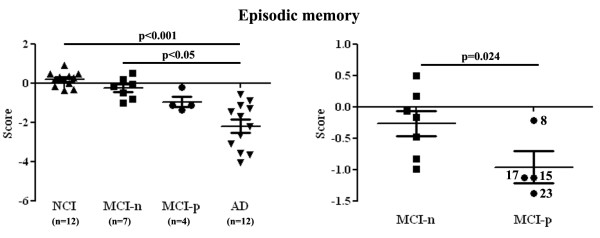
**MCI-p individuals presented intermediate deficits of episodic memory between those of AD cases and of NCI cases and MCI-n cases.** Left graph shows statistical comparisons of episodic memory scores between NCI, MCI-n, MCI-p and AD using Kruskal-Wallis test followed by Dunn multiple comparisons test. Right graph shows statistical comparison of same scores between MCI-n and MCI-p using Mann–Whitney test. Number of Subjects 8, 15, 17 and 23 is written beside each blot in right graph.

In secondary analyses, we evaluated the clinical and neuropathological features that distinguish individuals with MCI-p from the others. We analyzed the cognitive data and the concentrations of Aβ and tau of study participants (Table 1). Interestingly, the global cognition and episodic memory scores, the clinical hallmark of AD, were more impaired in AD compared to NCI and MCI-n, with MCI-p showing intermediate deficits. In the comparison of episodic memory scores between MCI-p and MCI-n, episodic memory scores of 3 individuals with MCI-p (Subjects 15, 17 and 23) were more impaired compared to all MCI-n subjects (Figure 4). Although a deficit of episodic memory in subject 8 was mild, deficits of episodic memory in some individuals of AD were also mild. By contrast, perceptual speed scores were lower in AD compared to NCI and MCI-p, with MCI-n showing intermediate deficits (Table 1). These analyses suggest that interaction of TDP-43 with p65 in neurons of temporal lobe occurred in a majority of MCI showing mild episodic memory deficits. The accumulation of Aβ42 and phosphorylated tau in the cerebral cortex was more prominent in AD compared to only NCI and the accumulation of insoluble tau in AD was higher than both NCI and MCI-p, whereas the accumulation of soluble and insoluble Aβ40 was lower in MCI-p, especially in subject 8 (98.2, 6.4, respectively) and 17 (65.0, 5.5, respectively), compared to NCI, MCI-n and AD (not significant). In CERAD, Braak and Regan score, the scores of individuals with MCI-p and MCI-n were intermediate level between AD and NCI. In the neuropathological score of hippocampal atrophy, the severity of the individuals with MCI-p was similar to AD compared to NCI and MCI-n (not significant). The accumulation of TDP-43 and p65, and the presence of phosphorylated p65 in TBS-soluble fraction were superior in MCI-p compared to NCI, MCI-n and AD.

**Table 1:** Selected characteristics of subjects from the religious order study with a clinical diagnosis of no cognitive impairment, mild cognitive impairment, or Alzheimer’s disease

**Characteristics**	**NCI**	**MCI-n**	**MCI-p**	**AD**	**Statistical analysis**
n	12	8	4	12	
Men,%	8.4	62.5	25.0	25.0	chi square test, *χ*^2^ = 7.13; p = 0.07
Age at death, mean ± SD, y	85.0 ± 6.0	85.1 ± 3.4	83.3 ± 4.6	86.1 ± 5.8	K-W test, p = 0.71
Education, mean ± SD, y	17.5 ± 3.9	19.4 ± 2.8	20.0 ± 1.6	18.0 ± 2.8	K-W test, p = 0.32
MMSE, mean ± SD	27.4 ± 2.0	26.6 ± 2.3	27.5 ± 1.9	16.2 ± 8.9^***, #, †^	K-W test, p < 0.0001
Time since last MMSE, mean ± SD, d	276 ± 327	256 ± 225	199 ± 117	281 ± 80	K-W test, p = 0.48
Global cognition score, mean ± SD	−0.12 ± 0.23	−0.32 ± 0.52	−0.64 ± 0.31	−1.75 ± 0.96^***, ##^	K-W test, p < 0.0001
Episodic memory, mean ± SD	0.18 ± 0.37	−0.27 ± 0.54	−0.96 ± 0.51	−2.19 ± 1.19^***, #^	K-W test, p < 0.0001
Semantic memory, mean ± SD	−0.37 ± 0.47	−0.20 ± 0.59	−0.43 ± 0.59	−1.47 ± 1.24^#^	K-W test, p = 0.015
Working memory, mean ± SD	−0.32 ± 0.42	−0.19 ± 0.71	−0.49 ± 0.62	−1.12 ± 0.88	K-W test, p = 0.046
Perceptual speed, mean ± SD	−0.29 ± 0.66	−1.06 ± 0.96	−0.27 ± 0.66	−2.08 ± 0.86^***, †^	K-W test, p = 0.0008
Visuospatial ability, mean ± SD	−0.44 ± 0.63	−0.10 ± 0.60	−0.44 ± 0.55	−1.37 ± 0.93^##^	K-W test, p = 0.0051
apoE e4 allele carriage,%	25.0	37.5	25.0	50.0	chi square test, *χ*^2^ = 1.87; p = 0.60
Cerebellar pH, mean ± SD	6.36 ± 0.31	6.43 ± 0.25	6.53 ± 0.11	6.49 ± 0.37	K-W test, p = 0.61
Postmortem delay, mean ± SD, h	7.4 ± 6.4	6.2 ± 3.6	5.7 ± 5.6	6.3 ± 3.9	K-W test, p = 0.90
Neuritic plaque counts, mean ± SD	2.3 ± 2.8	6.1 ± 7.0	1.8 ± 3.5	25.9 ± 26.5^**^	K-W test, p = 0.0054
Diffuse plaque counts, mean ± SD	12.3 ± 23.7	29.6 ± 29.5	9.0 ± 10.5	20.4 ± 17.2	K-W test, p = 0.11
Aβ_40_ concentration (soluble), mean ± SD	714.2 ± 1055.3	384.2 ± 685.0	111.7 ± 47.4	480.9 ± 588.0	K-W test, p = 0.48
Aβ_40_ concentration (insoluble), mean ± SD	1929.7 ± 3983.7	340.2 ± 672.2	42.7 ± 68.6	805.4 ± 2294.6	K-W test, p = 0.25
Aβ_42_ concentration (soluble), mean ± SD	2.5 ± 2.5	3.7 ± 3.1	1.8 ± 2.5	4.7 ± 2.9	K-W test, p = 0.10
Aβ_42_ concentration (insoluble), mean ± SD	1021.8 ± 1045.4	1312.4 ± 1445.6	749.1 ± 918.2	2523.2 ± 1609.9^*^	K-W test, p = 0.023
Neurofibrillary tangle counts, mean ± SD	0.5 ± 0.7	2.1 ± 4.8	1.5 ± 1.9	8.6 ± 15.0	K-W test, p = 0.29
Total tau content (soluble), mean ± SD	1.0 ± 0.2	1.1 ± 0.1	1.1 ± 0.3	0.9 ± 0.2	K-W test, p = 0.19
Total tau content (insoluble), mean ± SD	0.6 ± 0.2	1.1 ± 0.7	0.5 ± 0.1	1.5 ± 0.9^**, †^	K-W test, p = 0.0024
Total phospho-tau content (soluble), mean ± SD	0.0 ± 0.0	0.1 ± 0.2	0.2 ± 0.2	0.3 ± 0.4^*^	K-W test, p = 0.028
Total phospho-tau content (insoluble), mean ± SD	0.3 ± 0.2	0.9 ± 1.2	0.2 ± 0.2	2.1 ± 2.6 ^*^	K-W test, p = 0.029
CERAD score 4/3/2/1 (n)	3/3/5/1	3/0/3/2	3/0/1/0	0/1/3/8	n/a
Braak score I/II/III/IV/V (n)	2/0/6/4/0	0/0/3/4/1	0/0/2/2/0	0/0/5/1/6	n/a
Reagan score 3/2/1 (n)	7/5/0	3/4/1	3/1/0	1/5/6	n/a
Hippocampal atrophy score, mean ± SD	2.1 ± 1.7	2.4 ± 1.3	2.7 ± 2.3	2.7 ± 1.5	K-W test, p = 0.81
Full length (43kda) of TDP-43 content (TBS-soluble), mean ± SD	0.3 ± 0.2	0.2 ± 0.2	1.2 ± 0.7^#^	0.3 ± 0.3	K-W test, p = 0.043
Total p65 content (TBS-soluble), mean ± SD	0.4 ± 0.3	0.3 ± 0.3	1.0 ± 0.3^##^	0.4 ± 0.2	K-W test, p = 0.016
Presence of phospho-p65 content (TBS-soluble),%	0.0	12.5	75.0	8.3	chi square test, *χ*2 = 14.75; p = 0.0020

Intergroup comparisons: ^*^, p < 0.05 versus NCI; ^**^, p < 0.01 versus NCI; ^***^, p < 0.001 versus NCI; ^#^, p < 0.05 versus MCI-n; ^##^, p < 0.01 versus MCI-n; ^†^, p < 0.05 versus MCI-p.Aβ, beta-amyloid; AD, Alzheimer’s disease; CERAD, Consortium to Establish a Registry for AD; K-W test, Kruskal-Wallis test; MCI, mild cognitive impairment; MCI-n, MCI without interaction of TDP-43 and p65; MCI-p, MCI with interaction of TDP-43 and p65; MMSE, Mini Mental State Examination; NCI, no cognitive impairment; SD, standard deviation; TDP-43, Transactive response DNA binding protein 43.

We also compared the clinical and neuropathological data of individuals with AD-p and NCI-p with individuals without interaction (AD-n; Additional file 2: Table S1, and NCI-n). Hippocampal atrophy of the individual with AD-p (Subject 2) was prominent compared to AD-n. However, there were no significant differences in cognitive abilities and the concentrations of Aβ, tau, TDP-43 and p65 between AD-p and AD-n. Although the age of individuals with NCI-p (Subjects 7, 14, 21 and 29) were older than NCI-n (90.1 ± 4.6, 82.4 ± 4.9, respectively; p = 0.028), there were no differences in cognitive abilities and the concentrations of Aβ, tau, TDP-43 and p65 (data not shown).

## Discussion

In the present study, we identified a subtype of MCI (MCI-p) displaying nuclear interaction between TDP-43 and p65 NF-κB in the neurons from the temporal lobe (Figures 1, 2, 3 and Additional file 1: Figure S1). Both TDP-43 and p65 NF-κB levels and interaction of TDP-43 with p65 NF-κB in the temporal cortex were prominent in this subtype of MCI cases compared to AD and NCI individuals, as determined by immunoprecipitation assays and immunofluorescence microscopy. These findings are in line with the previous report of an upregulation and interaction of TDP-43 with p65 NF-κB in the spinal cord of ALS [23]. Since this TDP-43/p65 pathology was associated with deficits of global cognition and episodic memory in MCI (Figure 4 and Table 1) but not AD pathology, one may speculate TDP-43/p65 leads to higher risk of developing AD symptoms without aggravation of classical Aβ/tau neuropathologies.

TDP-43 cytoplasmic inclusions, which consist mainly of TDP-43 C-terminal fragments of ~25kD, were first described as pathological hallmark of ALS and FTLD-U cases [1,3]. The C-terminal TDP-43 fragments induced toxicity in cell culture systems [42-45]. However, neuronal overexpression at high levels of WT or mutant TDP-43 in transgenic mice caused a dose-dependent degeneration of cortical and spinal motor neurons without cytoplasmic TDP-43 aggregates [46-49] suggesting that an up-regulation of TDP-43 in the nucleus may also contribute to neurodegeneration. Cytoplasmic TDP-43 aggregates have also been detected in up to 75% of patients with a pathologic diagnosis of AD [8-12] as well as in the parietal lobe from MCI patients, with an intermediate level between AD and NCI [21,22]. However, compared to AD and NCI, our results indicated that levels of soluble full length of TDP-43 and p65 were the highest in a subset of MCI cases which exhibited an interaction of TDP-43 with p65 (Figure 1 and Table 1) and colocalization of TDP-43 with p65 in the nucleus of neurons (Figures 2, 3 Additional file 1: Figure S1). Although one individual with AD (Subject 2) displayed weak interaction of TDP-43 with p65 NF-κB by immunoprecipitation assays, this subject displayed low concentration of TDP-43 and p65 (Figure 1a, Additional file 2: Table S1) and low frequencies of colocalization of TDP-43 with p65 (Figure 3c). This suggests that neuronal dysfunction due to TDP-43-mediated NF-κB activation may occur in subgroup of MCI before progression towards AD. MCI refers to a transitional state between the cognition of normal aging and early dementia, especially AD [15]. Although not all MCI cases evolve to AD, impairment in episodic memory is most commonly seen in individuals of MCI who subsequently progress to AD [16,17]. The diagnosis of MCI due to AD, which includes amnesic form of MCI, is based on clinical and cognitive syndrome. Current biomarkers under consideration for AD and MCI are cerebrospinal fluid of Aβ and tau, PET amyloid imaging and hippocampal volume measure [17]. However, these markers are not strictly specific for AD, and there is no specific biomarker for MCI before progressing to AD. Here, 3 individuals with MCI-p (Subjects 15, 17 and 23, 75% of MCI-p subjects) presented intermediate deficits of episodic memory, which were more impaired compared to all MCI-n subjects (Figure 4 and Table 1). This suggests that the interaction of TDP-43 with p65 may represent a pathological biomarker for MCI due to AD, which is supported by clinical and cognitive syndrome. Although a deficit of episodic memory in subject 8 was mild, some individuals with AD also exhibited mild deficits of episodic memory (Figure 4). Therefore, the possibility of Subject 8 being a MCI due to AD cannot be excluded with only one point of episodic memory deficit.

Neuropathology analysis in Table 1 shows that the accumulation of Aβ42 and phosphorylated tau in the cerebral cortex is more prominent in AD compared to NCI and the accumulation of insoluble tau in AD was higher than both NCI and MCI-p, whereas the accumulation of Aβ40 is lower in MCI-p compared to NCI, MCI-n and AD. Lower accumulation of insoluble tau in MCI-p suggests that MCI-p is an early pathological phase of AD. In the Aβ pathology of FTLD, diffuse Aβ42 containing plaques were observed in the frontal cortex of 26% subjects with few neuritic Aβ40 plaques [50] and Aβ deposition generally appears to be commensurate with the age of individuals [51-53], which is difficult to distinguish with Aβ pathology in MCI [18-20]. However, TDP-43 pathology in same series of samples [21] is not a typical pathology as FTLD-TDP [2,40,41]. There was cytoplasmic detection of TDP-43 in neuronal subsets of these MCI samples but without TDP-43 inclusions like those found in FTLD-TDP. The accumulation of TDP-43 and p65, and the presence of phosphorylated p65 in TBS-soluble fraction were superior in MCI-p compared to AD, MCI-n and NCI. Although soluble aggregates of Aβ40 can trigger activation and nuclear translocation of NF-κB, high concentrations and long exposures of Aβ40 can reduce p65 NF-κB activation in neuronal cells [25-27]. Therefore, it seems reasonable that lower levels of Aβ40 is associated with higher accumulation of p65 in MCI-p and that higher accumulation of Aβ40 is associated with lower accumulation of p65 in AD. The activation of p65 NF-κB in MCI-p subtype may be induced either by the interaction of TDP-43 with p65 NF-κB [23] or low concentration of Aβ40. An upregulation of TDP-43 expression may increase the activity of BACE1 enzyme, thereby accelerating Aβ production [14]. Expression of p65 NF-κB increases BACE1 activity and APP processing [28,29]. In the progression of MCI-p toward AD, an upregulation of both TDP-43 and p65 NF-κB expression may increase the activity of BACE1 enzyme, thereby accelerating Aβ production, which in turn will induce the reduction of p65 NF-κB activation through progression toward AD. TDP-43 upregulation in MCI-p subtype may enhance to activation of NF-κB pathway [23] before progressing to AD. High concentration of p65 and TDP-43 and low concentration of Aβ40 in subject 8 may suggest the possibility of accelerating Aβ production and progression toward AD in future, even if this individual showed mild deficit of episodic memory at death. NF-κB signaling plays an important role in gene regulation involved in innate immunity, cell survival and inflammation [54,55]. It has been suggested that interactions of NF-κB with other protein molecules through the transactivation domain [56-58] could play an important part for gene regulation, in addition to the nuclear translocation and DNA binding of NF-κB [59,60]. Inhibition of NF-κB signaling reduced inflammatory processes and Aβ production in vitro and in vivo [61-65]. Therefore, the NF-κB pathway may be a key therapeutic target for both Aβ pathology and TDP-43 proteinopathy. It should be noted that MCI subjects exhibiting interaction of TDP-43 with p65 did not all exhibit phosphorylated p65 at ser536 (Figure 1). Although activation of NF-κB is also associated with phosphorylation of p65 [39], phosphorylated p65 can be found in both nucleus and cytoplasm [66,67] and phosphorylation site of p65 remains controversial [68]. It remains unknown to what extent interaction of p65 with TDP-43 requires selective phosphorylation at ser536.

Our results showed the accumulation of p65 and the presence of phosphorylated p65 at ser536 in a subgroup of persons with MCI (MCI-p) (Figure 1, Table 1). These individuals did not show higher Aβ42 or tau neuropathologies but the majority of them displayed cognitive impairment, including episodic memory deficits, at an intermediate level between NCI and AD (Figure 4 and Table 1). Based on data generated in the ALS field [23], these results suggest that inhibitors of NF-κB activation should be considered for treatment of MCI subtype with episodic memory deficits to prevent the developing AD and that potential therapeutic window would lie before their progression to AD. The number of subjects in our study was small and therefore further study with additional numbers will be needed to confirm this hypothesis.

## Conclusions

We propose that enhanced NF-κB activation due to TDP-43 and p65 interaction may contribute to neuronal dysfunction in MCI individuals with episodic memory deficits. Accordingly, treatment with inhibitors of NF-κB activation may be considered for MCI individuals with episodic memory deficits before their progression to AD.

## Competing interests

The authors declare they have no competing interest.

## Authors’ contributions

YO, JAS, DAB, FC and JPJ designed the study. YO and CT made the assessments. YO, CT, JAS, DAB, FC and JPJ drafted the manuscript. All authors contributed to the manuscript and approved the final manuscript.

## Supplementary Material

Additional file 1: Figure S1 TDP-43 colocalizes with p65 in the neuronal cells from the temporal cortex of individuals with MCI-p. **a-c** Sections from the temporal cortex of MCI-p (**a**, Subjects 17 and 23), AD without the interaction of TDP-43 with p65 in coimmunoprecipitation experiments (Figure 1B,C; AD-n, **b**, Subjects 19 and 28) or NCI-p (**c**, Subjects 14 and 29) were incubated with anti-TDP-43 and anti-p65 antibodies and subsequently with corresponding Alexa 488 and 633 antibodies, and imaged by confocal laser microscopy. Autofluorescence was detected using 575–630 nm bandpass emission filter. Arrows indicate the nuclear TDP-43 positive cells colocalized with p65 (**a**). Arrowheads indicate the nuclear TDP-43 positive cells without colocalization with p65 (**a-c**). Scale bars, 50 μm. Click here for file

Additional file 2: Table S1 Selected characteristics of subjects from the religious order study with a clinical diagnosis of Alzheimer’s disease. Click here for file
