# Supplementary figures and images for: Interaction of transactive response DNA binding protein 43 with nuclear factor κB in mild cognitive impairment with episodic memory deficits
Source: Acta Neuropathol Commun. 2014 Apr 1;2:37. doi: 10.1186/2051-5960-2-37 (PMC4230634; doi:10.1186/2051-5960-2-37)

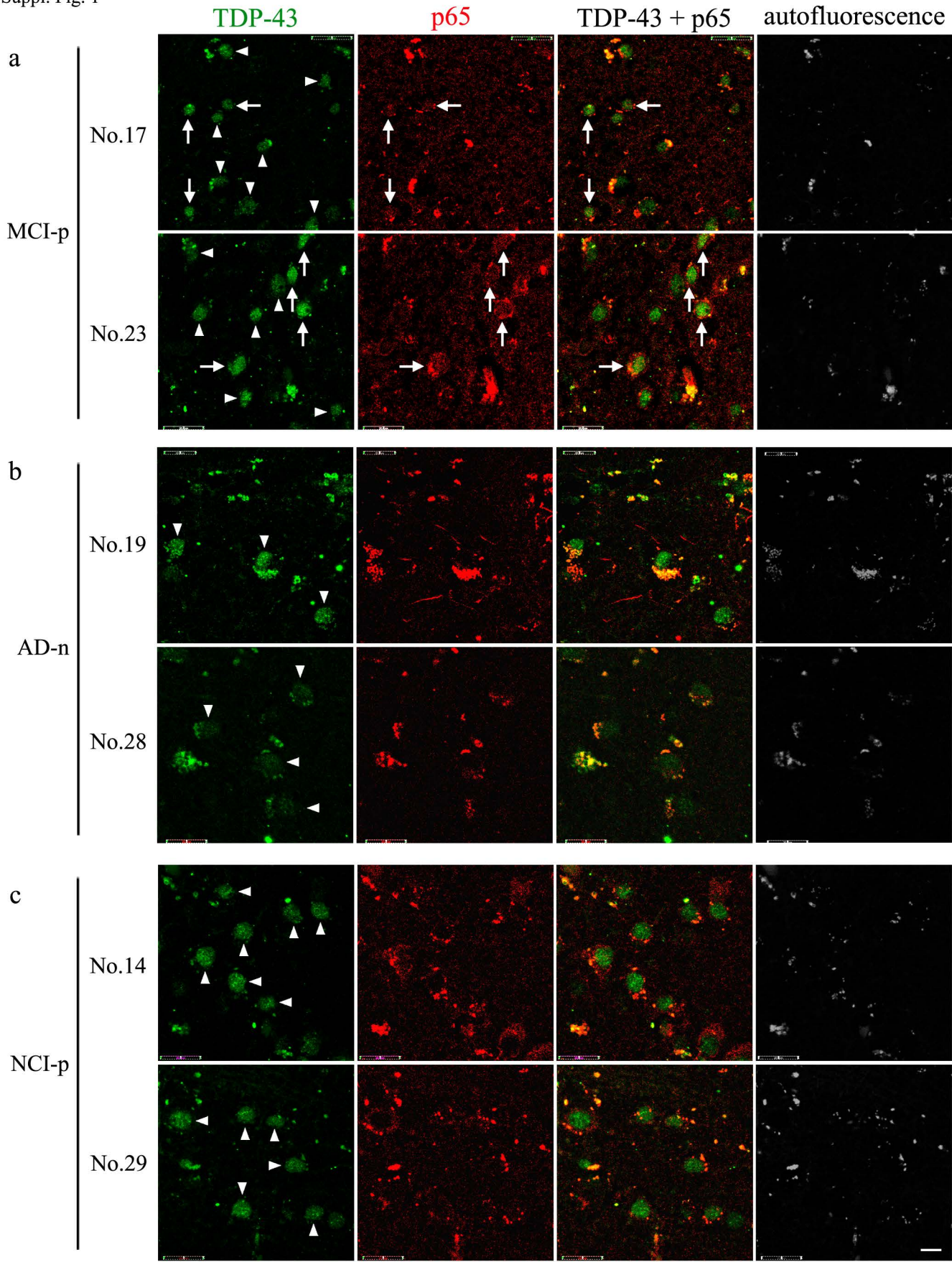

Supplement: Additional file 1: Figure S1 — TDP-43 colocalizes with p65 in the neuronal cells from the temporal cortex of individuals with MCI-p. a-c Sections from the temporal cortex of MCI-p (a, Subjects 17 and 23), AD without the interaction of TDP-43 with p65 in coimmunoprecipitation experiments (Figure 1B,C; AD-n, b, Subjects 19 and 28) or NCI-p (c, Subjects 14 and 29) were incubated with anti-TDP-43 and anti-p65 antibodies and subsequently with corresponding Alexa 488 and 633 antibodies, and imaged by confocal laser microscopy. Autofluorescence was detected using 575–630 nm bandpass emission filter. Arrows indicate the nuclear TDP-43 positive cells colocalized with p65 (a). Arrowheads indicate the nuclear TDP-43 positive cells without colocalization with p65 (a-c). Scale bars, 50 μm. [file 2051-5960-2-37-S1.pdf]
